# Harvard Odontological Society

**Published:** 1894-12

**Authors:** 

**Affiliations:** Harvard Odontological Society


### HARVARD ODONTOLOGICAL SOCIETY.

THE regular monthly meeting of the Harvard Odontological Society was held at Young's Hotel, Boston, on Thursday evening, April 26, 1894, at six o'clock, President Eddy in the chair.

A paper, entitled "Some Details as to the Care of Dental Instruments," was read by Dr. William H. Potter.

(For Dr. Potter's paper, see page 754.)

### DISCUSSION.

*President Eddy.*—It is with great pleasure that we have listened to this valuable paper from Dr. Potter; it is one of the most important subjects that we have had brought before us,—the cleanliness of our instruments.

*Dr. Hitchcock.*—I can emphasize what Dr. Potter has said most surely. The strength of modern surgery is the use of antiseptics; the only difficulty lies in what antiseptic to use and the method of using it. Formerly, consumption was understood to be non-contagious unless the patient was more or less predisposed. This theory is almost entirely exploded, and it is now conceded that the children of consumptive parents need not necessarily have consumption; the difficulty lies in the sputum. In Philadelphia a society has been formed whose object is to prevent expectorations in public places. They hold that a lady passing along the street takes up sputa upon her skirts, that it dries, and is blown through the air and inhaled. In Paris a family moved into a house which had previously been occupied by a consumptive. Two of the family who were perfectly healthy before contracted the disease. An examination proved that bacilli of tuberculosis were upon the walls; the moving of furniture, dusting, etc., put the germs into circulation, and they were taken into the lungs. Nature has a very kind way, if we are in a perfectly healthy condition, of protecting us from the action of these germs by throwing a deposit around them. Very frequently they are taken into the lungs and held there until a favorable opportunity is presented for them to commence their work. The amount of bacilli ejected in the sputa of consumptive patients varies from two hundred and fifty thousand to four millions in twenty-four hours.

In Europe about one million die every year of consumption, or one-eighth of all deaths. Probably nine-tenths of the cases of tuberculosis are caused by inhalation. Another method of infection is through wounds. Tuberculosis of the lungs will often be carried to the intestines and bowels by swallowing the sputa. Two cases of diphtheria were discovered in a Brockton school which were traced to the use of a lead-pencil. Syphilitic and diphtheritic cases may not exhibit marked symptoms. We may have a case of diphtheria where the person is not specially well, the throat not sore enough to be noticed, still, he can inoculate fatally another person. A case is related where a teacher going away for the summer kissed her pupils good-by at the train. Over half of them came down with diphtheria. A physician, a patient of mine, while attending a child with diphtheria, was bitten on the finger. He paid no attention to it at the time Soon, however, the glands of the axilla became very much swollen, the disease nearly proving fatal. Necrosis of the jaw followed, with loss of three or four teeth and quite a little of the bone.

Syphilis is commonly brought about by direct contact. France has passed a law requiring every workman to have his own blowpipe. Toy balloons with a whistle should not be used by our children; the vendor generally tries them first.

As to the frequency of these syphilitic mucous patches which were shown to us by Dr. Whitney two or three months ago, Vasserole in his investigations found that out of one hundred and thirty cases in men these mucous patches appeared on the tonsils in one hundred, on the lips in fifty-five, and on the tongue in eighteen. In women he found only about one-half this number had patches on the tonsils, attributing the cause in men to the use of pipes, chewing tobacco, etc., thus irritating the mouth.

I have lately had two or three cases of canker in the mouth that looked so nearly like those that Dr. Whitney showed us that special care was given to the instruments, the mirrors being labelled and set aside. Cases of tonsillitis, consumption, etc., have been treated in the same way.

The surgeons of to-day take no chances,—if they happen to drop a needle, for instance, on the floor, it is not used, because they make it a point to handle nothing but what has been thoroughly sterilized. It is because of this carefulness that surgery has become what it is,—an exact science. The cases of recovery after the operation of ovariotomy until this sterilizing precaution was adopted were very rare.

An article recently stated that a church in the western part of New York had employed an expert to examine the communion cup, and he found twenty-two varieties of bacteria, and they now use the individual cups. A church in West Roxbury has followed their example. The general idea is erroneous that syphilis is found principally among the lower classes.

I think the dentists themselves are in more danger of inoculation than their patients.

*Dr. Upham.*—We have had altogether in our meetings a good deal to say about the mouth-mirror and its sterilization; I would like to ask Dr. Potter how he takes care of it?

*Dr. Potter.*—I distinctly said in my paper that a mouth-mirror was not subject to sterilization, and if one has a syphilitic or a tubercular patient, the only precaution which he can take is to have a separate mouth-mirror. It is the most difficult thing we have to treat; it cannot be satisfactorily sterilized,—it can be washed mechanically, but that is about all.

I should judge from some of the remarks made to-night that the method of sterilization which I have spoken of was deemed a very laborious and troublesome thing, something that would interfere with one's practice and take up a great deal of time, but it is not so with me. If you have duplicate instruments, you need not dread the expenditure of time at all. It is something that I give no thought to, because it goes on so naturally and readily. I treat every case as if it were possibly contagious, because there are many cases that are contagious and yet are difficult to detect; so I put them all in one category, and whenever an instrument is used it is sterilized, and then put back into the case.

*Dr. Cooke.*—I would like to ask Dr. Potter what he does with the wooden-handled instruments?

*Dr. Potter.*—I do not use them.

*Dr. Cooke.*—Another thing very difficult to sterilize is the sleeve of the hand-piece of the engine. I have two or three of the hand-pieces, and treat them by washing thoroughly in hot water and putting in bichloride. I might also say that every instrument I use goes into a tray before I receive the next patient, and is washed thorougly with soap and water, and then put into a weak solution of bichloride.

*Dr. Potter.*—I do not wish to leave the impression that my instruments are not washed. I believe that washing is the foundation of all antiseptic surgery. My instruments are first washed, and are then sterilized as an additional precaution.

*Dr. Taylor.*—I have a habit of using a scaler with a large wooden handle, because I like the handle, and I put that into the sterilizer. I also treat the hand-piece of the engine in the same way. Mine has a cone socket, which I unscrew, then sterilize the point and wash the handle the best I can.

It is impossible to sterilize the mouth perfectly, and if we put the rubber dam upon the teeth, the soft tissues are protected. The hard tissues are not very susceptible to the action of the germs, and the chances of contagion from them are very small.

*President Eddy.*—I would like to ask Dr. Potter if he washes his rubber dam thoroughly before applying it?

*Dr. Potter.*—No, I do not. Rubber dam is handled mainly by machinery, and cannot come very much in contact with unclean things.

My idea is to have a system as perfect as possible. I do not claim that it is possible to eliminate every and all forms of germs, but we must do the best we can and take few chances.

Henry L. Upham, D.M.D.,
*Editor Harvard Odontological Society.*

